# Implementing lateral flow devices in long-term care facilities: experiences from the Liverpool COVID-19 community testing pilot in care homes— a qualitative study

**DOI:** 10.1186/s12913-021-07191-9

**Published:** 2021-10-25

**Authors:** Patrick Kierkegaard, Massimo Micocci, Anna McLister, John S. P. Tulloch, Paula Parvulescu, Adam L. Gordon, Peter Buckle

**Affiliations:** 1 NIHR London In Vitro Diagnostics Co-operative, Department of Surgery and Cancer, Imperial College London, St Mary’s Hospital, Praed Street, London, W2 1NY UK; 2 CRUK Convergence Science Centre, Institute for Cancer Research & Imperial College London, Roderic Hill Building, South Kensington Campus, Exhibition Road, London, SW7 2AZ UK; 3 Institute of Infection, Veterinary and Ecological Sciences, University of Liverpool, Liverpool, CH64 7TE UK; 4 Public Health Department, Liverpool City Council, Liverpool, Cunard Building, Water Street, Liverpool, L3 1DS UK; 5 Division of Medical Sciences and Graduate Entry Medicine, University of Nottingham, Nottingham, UK; 6 NIHR Applied Research Collaboration East Midlands (ARC-EM), Nottingham, UK

**Keywords:** Lateral flow devices, Antigen test, SARS-CoV-2, Nursing homes, Residential homes, Care homes

## Abstract

**Introduction:**

Antigen-based lateral flow devices (LFDs) offer the potential of widespread rapid testing. The scientific literature has primarily focused on mathematical modelling of their use and test performance characteristics. For these tests to be implemented successfully, an understanding of the real-world contextual factors that allow them to be integrated into the workplace is vital. To address this gap in knowledge, we aimed to explore staff’s experiences of integrating LFDs into routine practice for visitors and staff testing with a view to understand implementation facilitators and barriers.

**Methods:**

Semi-structured interviews and thematic analysis.

**Results:**

We identified two main themes and five subthemes. The main themes included: visitor-related testing factors and staff-related testing factors. Subthemes included: restoring a sense of normality, visitor-related testing challenges, staff-related testing challenges, and pre-pilot antecedent factors.

**Conclusion:**

Our study demonstrates that the real-world implementation of LFDs to test visitors and staff faces significant challenges as a result of several contextual factors negatively affecting the work practice and environment. More comprehensive studies are needed to identify and inform effective implementation strategies to ensure that LFDs can be adopted in an agile way that better supports an already exhausted and morally depleted workforce.

## Background

Care homes have been disproportionately affected by severe acute respiratory syndrome coronavirus 2 (SARS-CoV-2), with deaths in care homes estimated to account for over half of the total COVID-19 mortality in the United Kingdom (UK) [1]. Given that residents in care homes are at a significantly higher risk of experiencing severe disease and death due to SARS-CoV-2 [2], strict infection control measures were adopted, particularly indoor visitor restrictions (e.g., family and friends), to protect residents and prevent the introduction of the virus. However, this approach has led to residents becoming socially isolated [3], with increased risk of cognitive and physical functional decline [4–7]. Isolation undermines emotional connections between family caregivers and residents and may usurp fundamental human rights [8, 9].

Antigen-based lateral flow devices (LFDs) have been proposed by the UK authorities as a way of rapidly identifying and preventing infectious asymptomatic individuals homes who have COVID-19 from entering homes. They can be conducted without the need for specialised laboratory-based testing equipment, specifically polymerase chain reaction (PCR) tests. It has been asserted that this approach could be used to ‘test-to-protect’ vulnerable people and settings (for example, people living in care homes) [10, 11]. LFDs address some of the limitations of PCR-based testing protocols issued by the UK government for care homes. Particularly, LFDs are not subject to the same resource limitations and, hence, test delays that have been experienced with PCR tests, which has led to them being unable to break transmission chains and prevent outbreaks in care homes due to delays in identifying and reporting positive cases [1].

Most of the evidence generated surrounding the application of LFDs has primarily focussed on test performance versus PCR, and mathematical modelling [12–16]. This is important research but overlooks complex real-world factors that may hinder test utilisation and performance in complex work environments such as care homes [17–20]. The practical and emotional work associated with the pandemic [21, 22], could lead to substantial divergence between laboratory and real-world performance [23]. Numerous contextual issues could lead to low implementation uptake, compounding any diagnostic limitations of LFDs [24, 25].

Considering the vulnerability of care homes to outbreaks, it is crucial to explore the barriers and facilitators that influence implementation of LFDs into care homes. Eliciting staff perspectives can guide implementation efforts by taking into account real-world contextual factors which influence the ability of staff to effectively integrate lateral flow testing into existing organisational structures and workflows.

In the present study, we aimed to explore care home staff’s experiences of integrating LFDs into routine practice for visitor and staff testing, with a view to understanding implementation facilitators and barriers.

## Methods

### Design

A qualitative methodological approach was applied to enable us to explore the views of staff working at nine care homes concerning their experiences of integrating LFDs into their routine practice for visitor and staff testing. As the focus of this study was to neither form or explore complex theory but rather to better understand the barriers and facilitators experienced by care home staff, we adopted a qualitative approach [26]. Data were collected using in-depth interviews to elicit study participants’ viewpoints and experiences, and thematic analysis was used to identify key themes. We used the consolidated criteria for reporting qualitative research (COREQ) to inform our reporting of this study [27].

### Setting

The study took place in December 2020 in the North-West of England as part of the Liverpool COVID-19 community testing pilot, which offered people living and working within Liverpool City access to asymptomatic testing services [28]. The community testing pilot was initiated by Liverpool City Council, NHS Test and Trace (Department of Health & Social Care), NHS Liverpool Clinical Commissioning Group, Cheshire & Merseyside Health & Care Partnership, and The University of Liverpool (evaluation partner) ahead of the rest of the UK. This study contributes to the findings of the community testing pilot by focusing specifically on the use of LFDs in care home settings.

The testing pilot, originally called MAST (Mass, Asymptomatic, Serial Testing), used the Innova SARS-CoV-2 lateral flow device (Innova Medical Group, USA) to support the large-scale testing of people living and working in Liverpool. A tailored approach, known as the SMART (Systematic, Meaningful, Asymptomatic, Repeated testing), programme was used to guide the testing strategy. The SMART programme comprised three components: test-to-protect, test-to-release, and test-to-enable [29].

Care homes followed the test-to-protect component of SMART, which involved a protocol that required care home staff to undergo repeated testing, comprising two LFD and one PCR testing on a weekly basis. Weekly PCR testing was already part of the established guidelines published by the UK Department of Health and Social Care and the SMART programme introduced twice weekly LFDs over and above this [30].

Visitors followed a testing protocol that required one LFD and PCR to be undertaken at an asymptomatic testing site within the 24 h before their scheduled visit, followed by a second LFD performed by staff upon their arrival at the care home [31].

### Recruitment and participants

The recruitment of care homes occurred between November–December 2020. Purposive sampling was used to identify and recruit study participants and to ensure that a broad representation of staff working in a range of care homes (residential and nursing homes) were included in the study. To ensure the reliability and quality of the data gathered, we applied inclusion criteria where we sought to interview staff who had received training on how to use the LFDs, were directly involved in working with the LFDs to administer visitor and staff testing, and had been working at the care homes prior to the first national lockdown in March 2020, with the rationale that such staff would have a longitudinal perspective from working before and during the pandemic, on how different testing regimes had influenced care home work, and vice versa.

As part of the recruitment strategy, members of the study team initially attended three meetings arranged between Liverpool City Council and representatives from several care homes who had expressed interest in participating in the COVID-19 community testing pilot for care homes. During these meetings, members of the study team provided a brief introduction of themselves, explained the intent of study, and addressed any questions raised concerning the nature of the study. Following this, Liverpool City Council contacted the care homes to invite them to take part in the study, which resulted in a total of nine care homes (four nursing homes, and five residential homes) agreeing to participate in this study. Willing care homes were then contacted by members of the research team who emailed the care home managers to obtain their assistance in helping identify and recruit relevant staff members to interview for the study. Staff members who fitted the study eligibility criteria were approached by email and provided with a participant information sheet and asked to complete and return a consent form, as well as provide times convenient to them to be interviewed.

Recruitment stopped when the study team determined that data saturation had been achieved after interviewing 12 participants as no new themes were emerging from the interviews [32]. To remove doubts, we conducted three additional interviews to confirm that the data saturation had been reached.

### Data collection

All data collection took place between December 2020 and January 2021. A semi-structured interview guide was used to structure the interviews and was used flexibly to ensure both that the key questions concerning the study objectives were covered and that participants were able discuss areas of importance to them. All interviews were conducted by three members of the research team (two males and one female) with experience in qualitative methods and backgrounds in health services research (PK), human factors engineering (MM), and biomedical engineering (AM).

Before commencing the interviews, participants were asked if they had any questions regarding the purpose of the study and were provided with a summary of the study aims if they required further explanation. All interviews were conducted remotely and recorded using MS Teams video conferencing software. All the participants agreed to being recorded. Following the conclusion of the interviews, recordings were transcribed verbatim using the Otter.ai transcription software and were anonymised. Participants were not financially rewarded for participating in this study. Interviews lasted 30–45 min.

### Interview topic guide

The interview guide consisted of questions that began with general questions before focusing on the study’s key objectives of exploring staff members’ experiences of working with the LFDs and their attitudes towards integrating these devices into their workflow. The development of the interview guide was informed by prior research conducted by members of the study team on SARS-CoV-2 testing [33, 34], and informal discussions with staff from various care homes to identify points of criticality which had a bearing on the adoption of LFDs. We piloted the guide with a clinician who has experience working with medical diagnostics and this aided refinement of our questions concerning device usability. Following this, the interview guide was reviewed independently by two human factors consultants and a clinician with expertise in medical device research who were not part of the study team (see Acknowledgements). A clinician researcher in gerontology (ALG) then reviewed the questions and in the last stage we consulted with a patient involvement group (described further below) to make the final refinements to the interview topic guide to ensure validity and understandability of the study protocols.

### Data analysis

The data were subjected to thematic analysis [35], and coding took place between December 2020 and February 2021. All transcripts were coded using NVivo 1.3 software (QSR International). All transcripts were read and re-read independently by three members of the research team (PK, MM, AM) to gain a rich and deep understanding of the data and minimise researcher bias. PK developed the first iteration of the code book using an open coding technique and kept a reflexive diary to explore the interpretation process to generate the initial list of codes [36]. Focused coding was then used to synthesise the initial codes to develop broader themes, and explore the relationship between the different initial codes, where they were grouped together into meaningful categories to form overarching themes and subthemes. MM and AM independently reviewed the code book to identify missing themes and themes not adequately supported by the data. To ensure rigour, the team continually updated the code book and engaged in peer debriefing on a weekly basis with the wider study team to discuss any new emerging themes, review and reconcile the coding for any discrepancies, share preliminary data interpretations, and refine the thematic structure and hierarchy for the code book. At least two members coded each transcript (double coding), and “member checks” were conducted where results were presented to the care home managers at a meeting in February 2020 to confirm the themes identified and ensure the credibility and trustworthiness of the study findings. Coding continued until no new themes emerged from the data analysis (saturation) [37].

### Patient and Public Involvement

The study design and protocol was reviewed by members of the CONDOR Patient and Public Involvement (PPI) panel [38]. The CONDOR PPI panel comprises public lay members who have experience in the field of diagnostics for SARS-CoV-2 tests. The group provided input into the research prioritisation questions and provided specific input to the interview topic guide. All suggestions for amendments were incorporated into the final version of the study protocol.

## Results

### Characteristics of study sites and participants

In total, interviews were scheduled with 15 staff members (1 male, 14 female) from nine care homes. Of the 15 members, 11 were managers or supervisors, one was a senior carer, two were staff nurses and one was an administrator who was also designated as “COVID lead” for the home. Table 1 provides an overview of the site and participant characteristics.

**Table 1:** Participant and Site characteristics

**Site characteristics (*n*** **= 9)**		
**Type of care homes**	**Number**	
Nursing homes	4	
Residential homes	5	
**Number of staff**	**average (min, max)**	
Nursing homes	33 (20, 50)	
Residential homes	38 (26, 48)	
**Number of residents**	average (min, max)	
Nursing homes	32(18,46)	
Residential homes	45(27,56)	
**Care Quality Commission (CQC) rating domain**	**Percentage and number of care homes in each CQC rating for care homes in Liverpool City Council (LCC) and England**	
	**LCC**	**England**
Inadequate	1.3%	1.4%
Requires improvement	18.2% (3)	16.0%
Good	79.2% (6)	78.2%
Outstanding	1.3%	4.4%
**Participant characteristics (*n*** **= 15)**	**Number**	
**Gender**		
Male	1	
Female	14	
**Job role**		
Managerial	11	
Senior carer	1	
Staff nurse	2	
Administrator	1	

### Themes

Two main themes and five subthemes were derived from the analysis of the transcripts. The main themes included: visitor-related testing factors and staff-related testing factors. Subthemes included: restoring a sense of normality, visitor-related testing challenges, staff-related testing challenges, and pandemic induced work stressors (Table 2). Together these themes reflect key elements that affect staff’s perspective concerning the social and functional impact of LFDs. Representative quotations have been edited to correct grammar.

**Table 2:** Themes, subthemes, and description

Theme	Subthemes	Description
1. Visitor-related testing factors	Restores a sense of normality	Situations where staff viewed LFDs as a means for promoting restoration relationships and social support between residents and their families.
	Unaccounted work and environmental factors	Workload challenges of integrating visitor testing in terms of additional time spent and difficulties with scaling-up visitor testing.
2. Staff-related testing factors	Testing regime and real-life external variables	Scenarios where the prescribed LFD testing protocol requiring frequent staff testing was incompatible with the staffs’ actual work schedule.
	Implications of test result	Concerns about the accuracy of the LFD and the implications it could have on income and workload.
	Pandemic induced work stressors	Contextual factors influencing staff members mental well-being in connection with the willingness to implement more changes into the work environment.

### Theme 1: visitor-related factors

Visitor-related factors consists of themes that focus on the impact LFDs have had on the resident’s mental-wellbeing and staff workload.

#### Restores a sense of normalcy

This subtheme evolved as staff members described the benefits they observed in terms of how LFDs enabled residents and family members to meet important social needs. Particularly, they emphasise the importance of LFDs being able to restore relationships and social support. One staff member explained:

“It really benefits the residents and family members. You know, it opens up those doors again. Yeah, I think that’s an important message to bring through the emotional aspect for it.” (Participant 04)

Another staff member said,

“The residents can see their families. There’s no better feeling than when you haven’t seen each other for 10 months. It’s a long time.” (Participant 07)

Reflecting on this, there was a consensus that LFDs could help enable the return of important physical interactions between residents and their family in the future such as hugging and holding hands.

“I think that that moving forward is sort of some normalcy if you like, they can sit together in clumps again, they can hold hands, they can hold them without feeling guilty.” (Participant 10)

#### Unaccounted work and environmental factors

In this subtheme, staff members explained that the 30-min it took to get the test results was not reflective of the actual work and time required to facilitate visitor testing. Staff members explained that administering the test to visitors would require a significant amount of time investment to sanitise the facility.

“It’s half an hour for the testing, then it’s half an hour for the visit, and then it’s taking one and a half, probably two hours deep cleaning in the room. So just for a half an hour visit, you’re looking at like three hours, for two visits there six hours, just for two half an hour visits, so to carry on and to be safe as well.” (Participant 08)

For some, the physical infrastructure of the buildings also limited their capacity to feasibly scale-up visitations without compromising public health and social distancing measures. As one staff member explained,

“I don’t think we’d still be letting everybody in at the same time anyway, because it’s gonna be hectic, and we haven’t got that many rooms where we can segregate them to just have a family visit. So, it’s only going to be one family at a time. It’s going to be a long time before we let all the visitors back in at once.” (Participant 07)

The quotes in this theme demonstrate the direct social benefits of LFDs in supporting residents and families re-establish important bonds. At the same time, it raises several work-related challenges that testing has not considered, especially with regards to its impact on staff workload.

### Theme 2: staff-related testing challenges

Staff-related testing challenges consists of themes that shed light on the impact testing would have on staff members workflow, workload, and mental-wellbeing.

#### Testing regime and real-life external variables

Protocols for LFDs required that staff members undergo regular testing several times a week. However, this presented a significant challenge as the testing regimen was impractical considering the nature of their work schedule. That is, staff workers would have to visit care homes on their days off, which presented travel, time, and cost challenges that they were not being compensated for.

“They are not willing to come back and do the LFD test on a fourth day or a fifth day that we’ve been told to do it within. So not many staff been able to do it because of travel and the cost and then their time and obviously this time of the year everyone is busy. You know, they don’t want to spend all these hours to come in for the lateral flow test.” (Participant 12)

In relation, they also felt that it would be problematic to test staff members before the beginning of their shift. Specifically, they would have to arrive early without receiving any additional pay.

“We can’t all test before we start shift, it’s just not practically possible... asking people to come in early for no money all the time. It’s not acceptable. It’s not fair on people who are already on minimum wage to expect more and more from them.” (Participant 14)

Furthermore, ensuring that all staff members were tested was viewed as a challenge based on staff reflections regarding care homes existing struggles to meet the weekly PCR testing requirements issued by the Department of Health Social Care prior to participating in the pilot programme. One interviewee explained:

“Every staff member should be tested every week, but we don’t manage to capture every staff member every week. I’d say on average, we manage to capture about 75% of them staff each week.” (Participant 13)

#### Implications of test result

Several of those interviewed were concerned about reports of accuracy, concerning the LFDs. There was a particular concern about false-positive tests and its potential to reduce their already fragile workforce in the event that they ended up having to send ‘non-infectious’ employees home to self-isolate because of faulty test results.

“I was quite reluctant because there was a 50/50 chance of a false positive. And so, I was a little bit concerned about that, if we’re going to have this test, and we got positive results, they’re going to have to isolate. I’m going to lose a lot of my staff …. I’m sending staff home and having to pull my hair out and bring down for agency staff or get to staff to cover them.” (Participant 06)

Others mentioned that staff were worried about the financial consequences of the test. For instance, there was a general fear of losing income if the test results came back positive.

“They expressed that fear, “I don’t want to be tested and I’ve got to go home and you’ll, you’ll make me stay off work and I’ll lose money”. And you know that they were really worried about that.” (Participant 10)

#### Pandemic induced work stressors

This subtheme describes the mental and emotional burdens that staff members were already struggling with prior to the start of the LFD pilot study, which had implications on their willingness to adopt new practices.

Staff members expressed that they were already exhausted from having to take on several other tasks that did not traditionally fit within the role of their position. They conceded that they were mentally fatigued, implying that the acceptance of integrating LFDs would only add to their everyday stress.

“There’s a lot of extra workload because everything has to now be done remotely. (For example) you would normally have a social worker come in and do review. Before, social workers would sit in the office and go through the care documentation without taking any of the staff’s time. Now, we’ve had to use additional (staff) time for scanning emails and doing all of the documentation work of social workers. We have to do video calls to do the assessments that we (previously) wouldn’t sit in on. So, like some assessments can take two, two and a half, maybe three hours.” (Participant 13)

Summarising another example of the additional tasks they are struggling with, one staff member shared,

“The most traumatic thing was the staff having to hold a laptop over somebody who’s just passed away for somebody on the other end to verify their death and that was a lot of the stuff to deal with. It’s telemedicine because the GP wouldn’t come out. I think that has been the biggest upset because as a residential home there’s no nurses on duty here. It’s care assistants, and that’s been massive.” (Participant 11)

For staff members, this also meant that the LFDs helped reduce the additional emotional labour that they had to bear in regards to being extra attentive to meeting residents’ emotional needs because of visitor restrictions.

“We were struggling because our staff are working extra hard. We’re not only providing what we normally provide. We provide emotional support, but we’re (having) to provide more of that now.” (Participant 06)

Also, staff members were suffering from low morale. Several expressed that they felt unappreciated and that their current efforts within the care sector was going unnoticed.

“It’s a thankless job anyway. You know, I mean, we don’t do it for the money. I do it because I love it. Because they (residents) are my family and I love them … We’re constantly battling with people who think our job is an unskilled job. We go clapping for the NHS … but carers get forgotten.” (Participant 04)

Taken together, these themes illuminate important contextual challenges of implementing routine LFD testing on staff. Experiences of excessive stress and poor psychosocial state can negatively affect their desire to integrate testing into their daily work routine.

## Discussion

To our knowledge, this is the first qualitative study to explore the real-world experiences of LFDs used by staff in care homes as part of their daily workflow to facilitate routine visitor and staff testing. Through the interviews, staff members revealed that LFDs enabled the restoration of important socio-relational factors, whilst integrating testing introduced several implementation challenges. Particularly, our study suggests that the contextual disconnects between increased workloads, non-work-related factors, and prescribed LFD testing protocols need to be addressed to facilitate the successful adoption of LFDs into long-term care settings.

### Visitor-related factors

Our findings revealed the consensus among staff that that LFDs could ‘**restore a sense of normality for residents’**, which was characterised by their ability to help re-establish social relationships and social support. This perspective suggests the dual role of LFDs in directly protecting residents and indirectly influencing their psychological wellbeing. Existing work highlights the importance of these factors in the quality of life and overall psychosocial wellbeing of residents, as maintaining socially meaningful relationships with family and friends positively impacts health outcomes [39–43].

Staff also underlined that LFDs enabled the restoration of non-verbal communication (e.g., sharing physical space and engaging in physical touch), thereby lifting restrictions that would limit families from otherwise engaging in positive expressions of affection and nurturing important emotional connections [8]. Importantly, touch such as holding hands enables relationship building, decreases stress, and alleviates anxiety during stressful situations [44–47]. Such behaviours between families reflect a degree of intimacy developed over a lifetime that provides emotional security for residents [8, 48]. Thus, the use of testing has the potential to create a sense of normality that, if used effectively, might reduce relational disruptions, support the emotional well-being of residents and reduce the risks of social disconnect from the world outside care homes [49, 50]. However, the perceived sense of normality facilitated by LFDs may also elicit behavioural responses that underperceive risk estimation and adherence to protective behaviours [51]. That is, the perceived sense of normality within the workplace can lead to the desensitisation of risk and invite complacency as a result of habituation, ultimately compromising infection control and prevention measures [52, 53]. It is noteworthy that, since this work was undertaken, that the UK government released visiting guidance in March 2021 that suggests hugging should be minimised and emphasises the importance of social distancing [54]. Thus, whilst tests may enable visitors to re-enter homes, it will not have facilitated all the advances in human interaction that staff had hoped for and expressed in our interviews.

Despite the psychosocial benefits, our study also found that visitor-testing encompassed several **‘unaccounted work and environmental factors’** that could limit the capability of homes to enable visitations. Staff members explained that the perception of the test time does not take into account the additional time needed to follow strict infection prevention and control procedures when facilitating a visit. A test that appears to take 30-min to generate a result could potentially take up to 3 h of a staff member’s time. This contrasts with pre-pandemic work where staff averaged 10–30 min to clean rooms during their daily routine or 45 min for deep cleaning [55]. Preventing environmental contamination through infection control practices (e.g., deep cleaning and disinfection) required two-hour time investment as guidelines typically entail a two-step process of first using regular detergent, followed by disinfection with hospital-grade disinfectant [56]. Previous work found that cleaning tasks can lead to staff frustrations due to the redistribution of workload that takes time away from providing resident care [57]. Moreover, this amplifies the existing situation where inadequate staffing has meant that carers are already struggling to find time to provide direct care [58].

An additional factor raised included many care homes being in older buildings, which creates difficulties in following social distancing protocols, as these require sufficient space between individuals in common areas of the facility. More research is needed to investigate testing in relation to finding the balance between infection control and architectural design, including retrofitting existing facilities to accommodate testing and visitor demands [59].

### Staff-related testing challenges

The study has identified a disconnect between the prescribed **‘testing regime and real-life external variables’** for staff testing. Staff expressed frustrations regarding the inconvenience and lack of monetary compensation of having to test at their workplace or arrive earlier or stay later than their paid shifts to get tested. The requirement for staff to get tested multiple times a week was not compatible with the contextual realities of the working schedule of care homes employees. Staff were adamant that it was impractical and inequitable to expect to staff to stay longer than their shift or travel into the facility without pay to get tested. This implies that LFDs testing regimes designed to increase accuracy face significant barriers that could amplify existing frustrations with current employment practices contributing to work-related stressors [60, 61].

Related research has found that care assistants identified monetary needs as a critical factor motivating their reason to work in care homes and that this affects job satisfaction [62, 63]. Importantly, prolonged exposure to ‘unpaid’ situations could lead staff to feeling significantly devalued in a job where the pay rate and status is already viewed as unattractive, employment benefits is limited, and staff have little say in their working arrangements [64, 65]. Moreover, requiring staff to test outside their working hours can result in poor job satisfaction due to the perceived loss of autonomy and empowerment [66].

Since this work was undertaken, the UK government commenced a national programme of LFD testing, which has been met in most parts of the country by care home staff undertaking testing in their own homes [67]. The impact of this on concordance – both in terms of frequency of testing and adherence to testing protocol – has not been investigated. It is likely that some of the factors already mentioned will influence concordance. The Vivaldi study reported that infection rates were higher in homes where staff were not guaranteed sick pay if they took time off due to suspected COVID – thus fear over non-pay could significantly impact how staff engage in testing regimes [68].

Failure to address the disconnect between LFD testing regimes and the care home workforce risks increases in staff turnover and burnout [61, 69, 70]. It is critical that any implementation of routine LFD testing for staff addresses the structural problems with regard to pay and retention, especially for those in part-time and temporary positions and with flexible working schedules.

Another important finding of this study concerns the **‘implications of the result of the test’** as there were significant concerns around the consequences of diagnostic uncertainty for LFDs. When discussing the scenario of a false-positive test, it was apparent that care home managers were fearful about losing staff due to the mandatory self-isolation protocols. The implications of losing staff members to false-positive tests would further increase the strain on the existing staff, who were already been working at full capacity during the pandemic. This has a knock-on effect of reducing the amount of time an individual staff member can spend addressing the needs of individual residents [71].

For staff members, a positive test result (incl. False-positive) may have serious financial consequences, especially for care assistants, as they are already on a low-wage and may be financially vulnerable [71, 72]. As a large majority of the staff undertake multiple part-time jobs [73], the risk of loss of income from a positive test result would only further undermine their willingness to embrace LFD testing. Consistent with other findings, the loss of income can lead to a heightened risk of anxiety and depression due to quarantine measures [74]. Here, financial support measures (e.g., via access to paid sick or comprehensive health insurance) will be critical if staff must self-isolate if they test positive and increase staff willingness to adopt LFDs. Staffing support from health and social care partnerships will be important to mitigate the risk of understaffing [75].

We also noted that ‘**pandemic induced work stressors’** played a role in staff hesitancy to consider accommodating LFDs into their daily practice, especially as they were already facing emotionally and physically taxing job stressors introduced by the changes in their job role [76]. Staff are experiencing exhaustion from taking on heavier caseloads and learning new roles outside the scope of their training [21], together with feeling demoralised as a result of the lack of public recognition for their work in social care [21, 71]. For instance, visitor restrictions meant that staff have had to take on the additional emotional burden of work needed to compensate for the lack of social connection between residents and their families [77]. Frequent changes are associated with psychological uncertainty [78], and emotional exhaustion that can lead to negative health outcomes [79]. These imply that attitudes toward change will be negative as care home staff are likely to view the new work changes associated with testing as a perceived loss of resources [80].

The findings presented in this paper demonstrate the complexities of integrating regular LFDs into the work environment of care homes during pandemic times. A valuable finding was that staff testing protocols are incompatible with the current setup of the care home work practices. Assumptions about how others go about their work without a clear, realistic understanding of the actual work and work system can lead to problems in compliance and task performance [81]. This suggests the need for more human-factors studies to inform the implementation of tests into complex socio-technical settings. Similar work has not been undertaken for PCR tests and there is need for more human-factors studies to inform the implementation of these tests in the real-world context [82, 83].

### Strengths and limitations

This qualitative study is the first study to our knowledge that provides data on staff members experiences of using LFDs in long-term care facilities as part of routine practice in a systematic way. Evidence in this area of study is limited and their perspectives are even less examined. By using a qualitative methodology, we were able to identify important themes that may impede or facilitate the implementation of LFDs into long-term care facilities. Our findings may help improve future work in addressing visitor restrictions and staff well-being in care homes during national lockdowns.

The main limitations of this study are the small sample size of staff members who participated in this study. Our findings are based on nine care homes from the same geographical area. This may limit the generalisability of the results. More research with larger samples from other care homes in different regions is needed to validate our findings. Secondly, some data were collected during the final phase of the pilot study. Perspectives of those staff members may have changed after more extensive exposure. Thirdly, staff members interviewed were selected by care home managers. Although we informed interviewees that their answers were confidential, socially desirable answers could not be fully excluded and may have influenced their answers.

## Conclusion

The real-world implementation of LFDs provides a new possibility to opening up care homes to visitors and enabling the rapid testing of staff to support infection prevention and control efforts. However, we have shown that the reliance on integrating an LFD testing regime to routinely screen staff and visitors is challenging without taking into consideration several contextual factors challenging the work practice and environment. More comprehensive studies are needed to identify and inform effective implementation strategies to ensure LFDs can be adopted in an agile way that better supports an already exhausted and morally depleted workforce.

## Data Availability

All relevant data are within the manuscript.

## Abbreviations

CONDOR: COVID-19 National Diagnostic Research and Evaluation Platform
COREQ: Consolidated criteria for reporting qualitative research
LFDs: Lateral flow devices
PCR: polymerase chain reaction
PPI: Patient and Public Involvement
SMART: Systematic, Meaningful, Asymptomatic, Repeated testing
UK: United Kingdom
